# RF-Based UAV Detection and Identification Using Hierarchical Learning Approach

**DOI:** 10.3390/s21061947

**Published:** 2021-03-10

**Authors:** Ibrahim Nemer, Tarek Sheltami, Irfan Ahmad, Ansar Ul-Haque Yasar, Mohammad A. R. Abdeen

**Affiliations:** 1 Department of Computer Engineering, King Fahd University of Petroleum and Minerals, Dhahran 31261, Saudi Arabia; tarek@kfupm.edu.sa; 2 Department of Information and Computer Science, King Fahd University of Petroleum and Minerals, Dhahran 31261, Saudi Arabia; irfan.ahmad@kfupm.edu.sa; 3 Transportation Research Institute, Hasselt University, BE3500 Hasselt, Belgium; ansar.yasar@uhasselt.be; 4 The Faculty of Computer and Information Systems, Islamic University of Madinah, Madinah 42351, Saudi Arabia; mabdeen@iu.edu.sa

**Keywords:** radio frequency, unmanned aerial vehicles, machine learning, detection and identification

## Abstract

Unmanned Aerial Vehicles (UAVs) are widely available in the current market to be used either for recreation as a hobby or to serve specific industrial requirements, such as agriculture and construction. However, illegitimate and criminal usage of UAVs is also on the rise which introduces their effective identification and detection as a research challenge. This paper proposes a novel machine learning-based for efficient identification and detection of UAVs. Specifically, an improved UAV identification and detection approach is presented using an ensemble learning based on the hierarchical concept, along with pre-processing and feature extraction stages for the Radio Frequency (RF) data. Filtering is applied on the RF signals in the detection approach to improve the output. This approach consists of four classifiers and they are working in a hierarchical way. The sample will pass the first classifier to check the availability of the UAV, and then it will specify the type of the detected UAV using the second classifier. The last two classifiers will handle the sample that is related to Bebop and AR to specify their mode. Evaluation of the proposed approach with publicly available dataset demonstrates better efficiency compared to existing detection systems in the literature. It has the ability to investigate whether a UAV is flying within the area or not, and it can directly identify the type of UAV and then the flight mode of the detected UAV with accuracy around 99%.

## 1. Introduction

In modern society and starting from this century, Unmanned Aerial Vehicles (UAVs), or drones, are widely used around the world. The recent popularity of UAVs is primarily due to the progress in developing the precision sensors, such as gyroscopes and motion sensors, which are employed to guide, navigate and control the UAVs for tracking and observation purposes in the candidate region. As a result, UAVs are fairly inexpensive and cost-effective compared with other available infrastructures, such as traditional satellite-based systems, sensing, and traditional communication systems. UAVs were first used by the military especially in the photogrammetry and 3D scanning for tracking and surveillance purposes, as in References [1,2,3]. Then, different civilian applications, including UAV photogrammetry, were developed, due to their low operating altitude, which can provide a high space resolution data [4,5]. The relative low cost of the UAV-based systems in many areas has earned considerable success. UAVs are now used in multiple areas, including tracking, search and rescue tasks, package delivery, smart policing, video recording, and precision agriculture [6,7]. In view of emerging trends in UAV technologies, UAVs are expected to become an integral part in the modern technologies.

UAV complements the conventional satellite-based systems, or even replace them [8,9,10]. However, the ubiquity of UAVs creates such security and privacy concerns. The massive rise for the commercial UAVs presents many problems in the fields of airspace management, and health and personal data protection, given the huge potential to promote and achieve better economic growth [11]. In September 2017, an Army Chopper was hit by an unauthorized drone across one of the residential area [12]. A small UAV also crashed in January 2015 in the lawn of White House and created concern about the existing safety measures [13]. Due to their low visibility, UAVs often provide the ideal platform for illegal smugglers. For example, U.S. officials recently confiscated smuggled medicinal products by the Mexican cartels [14], and the police in China has exposed the illicit smartphone trade from Hong Kong to the mainland of China [15]. UAVs are also used by prisoners to move items inside and outside the prison [16]. Moreover, they introduced more critical challenges for the public and officials due to their ability to carry high explosive payloads.

In reality, following the rules of the UAVs is not an easy task. Most UAVs still have not been registered, and other UAVs cannot have a geofencing or the geofencing option can be easily turned off. Hence, there is a huge demand to detect directly the intruder UAV in the restricted area or recognize its presence and the operating mode in the forensics investigations. The optimal detection technique should warn people if unauthorized UAVs entered the restricted area at earliest stage. Then, countermeasures can be applied on the UAV intruders and the UAV owner can be monitored and identified afterwards. Therefore, it is extremely necessary to effectively detect the consumers of UAVs. In addition to detecting UAVs, it is extremely beneficial for forensics to recognize the operating mode of the UAV. indeed, the ability to recognize the operating mode of the intruder UAV allows investigators to recover accidents that can be used as court proof in cases and enable officers to better countermeasure or respond to different potential UAV accidents. Based on different examples that were mentioned earlier, it is a prime concern to identify and remove intruding UAVs before introducing any serious problems in the infrastructure. Hence, UAV detection has become a priority in the current research directions due to the urgent need for human health, privacy and security. Academia and industry have investigated this challenge thoroughly for the commercialization of intruding UAV-based systems. The autonomous detection, monitoring, and identification of UAVs is a highly important operational key in some market systems and architectures that were suggested by researchers. In general, detection approaches work based on Radio Frequency (RF) signals [17], RAdio Detection And Ranging (RADAR) [18,19,20], acoustic [21], Light Detection and Ranging (LiDaR) [22], or based on cameras (passive optics), along with computer vision techniques [23]. These detection approaches may be less successful in some realistic situations, particularly in a crowded urban environment by using only one of these sensors for detection. Radar signals may be obscured by walls and buildings and other barriers, which in urban environments are very common. Vision techniques cannot be used in the dark and Non Line-of-Sight (NLoS) scenarios for detecting the UAVs. The acoustic-based identification techniques can be interfered with the sounds of the atmosphere that might overshadow the sound created by the UAV [17,18,19,20,21,22,23]. Table 1 summarizes the pros and cons of these detection techniques.

The investigation of the resulted RF signals from the UAV controllers is a promising process for detecting UAV as discussed in Reference [24]. Indeed, the behavioral biometrics of the UAVs can be defined using Machine Learning (ML) techniques from the captured RF signals. These techniques are trained based on the raw data, which is coming from the RF signals when the UAV controller is managed by the authorized owner. In this way, numerous UAVs and the human controller on the ground can be identified. Although the biometrics compliance of the UAV operator is significant information in the detection and identification, it could be that it is little knowledge of behavioral metrics about the attacker. Therefore, the detection of the intrinsic signature of the UAV controller itself should be a priority to detect and identify any attacker UAV. Such signatures can be derived from the RF signals that are generated by the UAV controllers; it is also known as the RF fingerprints of the UAV controllers.

In general, different companies are using the UAV technology by distributing them in the sky for achieving a specific task. For security reasons, it is required to identify if there is a UAV in a specific region or not, specify the type of UAV, and the flight mode of the UAV from the radio frequency signals. Hence, this helps to identify if there is any UAV intruder in this region and decide the suitable action. In this research, we will design a simple detection approach based on the machine learning technology for detecting the presence of the UAV in known region, and then specify the type and the flight mode of the detected UAV based on the captured RF signals for that UAV. The scope of the work is detection and identification problem based on a public dataset. We designed an intelligent hierarchical approach to detect and identify the UAV, this hierarchical approach is constructed based on a set of ensemble learning classifiers. It consists of four classifiers and they are working in a hierarchical way to detect the presence of a UAV, the presence of a UAV and its type (Bebop, AR, and Phantom), and the presence of a UAV, its type, and its mode. The main contributions of this research are summarized as follows:

1. **Review** the intelligent detection and identification learning techniques in UAV technology, show the performance of these techniques in term of accuracy, complexity, advantages, and limitations.
2. **Propose** a hierarchical learning approach to solve the detection problem based on ensemble learning. In this approach, we attempt to minimize the cost of the whole approach in order to make it simple, light, and efficient.
3. **Compare** the proposed approach with other learning approaches in term of accuracy.

The remainder of this research is organized as follows. The related works are presented in Section 2. The methodology and the system model is given in Section 3. Section 4 introduces the proposed detection and identification approach, and the results are given in Section 5. Section 6 shows a comparison between our approach and other learning approaches that use same dataset. At last, Section 7 presents the conclusions and future works.

## 2. Related Works

In this section, we summarize some of the recent research studies that are related to UAV classification and detection problem. In general, most of the presented approaches employ RF sensing elements to collect RF signal between the controller and UAV. However, we classified the previous studies into three groups based on the extracted feature as follows:

### 2.1. Acoustic- and Sound-Based Feature

Al-Emadi et al., in Reference [25], introduced several methods for drone identification and detection, such as Recurrent Neural Network (RNN), Convolutional Neural Network (CNN), and Convolutional Recurrent Neural Network (CRNN). Such methods were used in the analysis and recognition of the special acoustic signatures of flying drones. In addition, Al-Emadi et al. were able to figure out a variety of conclusions from various experiments carried out throughout the paper; first of all, in terms of F1 score, recall, accuracy, training on CPU, and testing time for both drone detection and identification, the CNN technique outperformed both RNN and CRNN techniques in terms of all mentioned metrics. Furthermore, and provided that the difference in efficiency between CNN and CRNN is negligible, CRNN is considerably faster than CNN, and the results from the three algorithms are more reliable. Eventually, this research provided an open source repository of various drone audio clips for the research community for further studies.

Anwar et al., in Reference [26], implemented a new ML framework for Amateur Drone (ADr) detection and classification based on sounds, which can be identified from different bird, aircraft, and thunderstorm sounds in a noisy ambient. The Mel Frequency Cepstral Coefficients (MFCC) and Linear Predictive Cepstral Coefficients (LPCC) extraction techniques were applied for extraction the required features from ADr sound. Upon the feature selection, Support Vector Machines (SVMs) were used to correctly distinguish these sounds with different kernels. The experimental results proved that the SVM cubic kernel with MFCC beats the ADr detection cycle by achieving approximately 96.7% of its accuracy. The findings further reveal that the ML system is more than 17% better than the drone sound detection system based on similarity, which lacks the ML prediction.

### 2.2. Physical Characteristics-Based Feature

Unlu et al., in Reference [15], implemented an entire independent drone monitoring system using computer vision and RGB cameras with a new algorithm named by Target Candidates Track (TCT). This is a series of several policies that incorporate the movement signatures on static picture planes and the visual signatures on zoomed cameras to prioritize several applicants and provide an appropriately specified time frame for determining any potential risk. Unlu et al. also suggested a hybrid deep learning detection system for effective use of memory and time. The resulted frame from the zoomed camera in the tower is superimposed on the frame of the wide-angle static camera. The main goal is to ensure that a possible target will not be pursued with the spinning cameras for a long time when there is a false alarm like bird or an aircraft, since a true threat might be missing in this situation. They demonstrated a completely autonomous UAV detection system based on two cameras, one of them is mounted on a revolving platform, with a low-angle lens to investigate such flying objects.

Alipour-Fanid et al., in Reference [27], presented a UAV detection and operating mode recognition ML approach with minimum delay over the encrypted WiFi UAV traffic. This approach derived the main features from the packet size and the inter-arrival time and then adopted a weighted one norm regularization in the training phase taking into account the measurement time of different features. Hence, the collection of features and the optimization of the output was combined into a single objective function. In order to deal with the ambiguity of the packet inter-arrival time when calculating cost function, they used the Maximum Likelihood Estimation (MLE) for finding the arrival time of the incoming packets. They gathered a significant number of WiFi-encrypted traffic from eight forms of UAVs and carried out a thorough assessment of the suggested methods. The experimental data indicated that these methods identify and classify UAVs in 0.15–0.35 s with a precision of 85.7–95.2%. In LoS and NLoS links, respectively, the UAV detection range is within a sensing range of 70 and 40 m. The modes of UAV operations can also be accurately defined in the range 88.5–98.2%. The description of the operating mode demonstrated the cyber physical coupling properties of UAVs. Based on the coupling concept, the information given by UAV on the cyber component (WiFi traffic data) can be obtained from the physical/operating status/mode (WiFi traffic data).

### 2.3. RF-Based Feature

Al-Emadi and Al-Senaid, in Reference [26], proposed a drone detection technique using a CNN, where the resulted RF signals during the communication between the drone and the controller were collected in order to be investigated in the learning stage. The proposed technique was trained and tested using a public dataset that compromises the collected RF signals for three type of drones. Hence, experimental results showed better performance compared with other techniques using deep neural networks in term of identification and detection purposes. The results of this research have proven the validation of using CNN for detection the intruding drones, and they achieved around 99.7% accuracy for classifying the availability of drone and 88.4% for specifying the type of the detected drone.

Medaiyese et al., in Reference [28], presented a new ML approach for detecting the intruding drones within a specific region, it basically uses the low band part of the captured RF signals during the communication process between the drone and its controller. It seems that the low band has more information about the signal compared with high band part. The eXtreme Gradient Boosting (XGBoost) algorithm was used in the proposed approach to detect and identify the availability of the drone, the type of the detected drone, and its operational mode. The approach is based on solving three classification problems (e.g., drone or not, type of the drone, mode of the drone), and they used three XGBoost models in their implementation and achieved accuracy around 99.96% in detecting if there is a drone or not, 90.73% in specifying the type of the detected drone, and 70.09% for the mode of the detected drone.

A UAV multiphase identification and ML classification system was implemented by Ezuma et al., in Reference [29], to classify 17 UAV control units, in the existence of interference resulted from wireless technologies, i.e., WiFi and Bluetooth. There are two detectors in the UAV multiphase detection system. The first detector uses the naïve Bayes algorithm of a dual-state Markov model to determine whether the collected data includes RF signals or not. The second detector decides, once the RF signal is detected, whether it is a UAV control or a source of interference. As the RF signal is sensed and resulted from an interfere source, WiFi or Bluetooth source is selected as the source class. On the other hand, the signal is sent to the ML classification system if the sensed signal resulted from the UAV controller in order to determine the UAV model and the make of the controller. K-nearest neighbor classification with 25 dB SNR reached a designation precision of 98.13%.

Shorten et al., in Reference [30], implemented a research to identify the location of the drone controllers from the RF signals. The signal spectrum is measured by RF sensor array. In order to predict the controller output relative to the sensor, a CNN was trained to predict and visualize this output. From these bearings, the location of the controllers can be determined given that at least two sensors are used within a reasonable separation distance.

Ezuma et al., in Reference [31], identified and distinguished micro-UAVs using RF signals sent from the device to a micro-UAV. This method starts by converting the time domain signal into energy time-frequency and determines the energy trajectory. A variety of statistical features are then derived from the energy rather than the time. With the help of Neighborhood Component Analysis (NCA), the dimensionality of the feature set is minimized; the main features are categorized using several ML approaches. The discrimination features can still be derived even if the signal in the time domain is added with some noise. In order to delete the bias and reduce data size, RF signals are converted into a wavelet domain. The naive Bayes method is then used to distinguish noise from micro-UAV signals based on the Markov models.

Al-Sa’d et al., in Reference [32], demonstrated a sophisticated algorithm for the detection and identification of intruding drones using RF database. The availability of a drone, the availability of drone and its form and, ultimately, the availability of a drone, form, and flight mode are observed by using three Deep Neural Networks (DNN). The efficiency of each DNN is measured and tested using different methods and validated using a 10-fold cross validation stage. The findings of classification revealed a decrease in the system efficiency as the number of classes rises. The accuracy of the first DNN (2 classes) has fallen from 99.7% to 84.5% in the next DNN (4 classes) and eventually to 46.8% in the third DNN (10 classes). The results of the proposed method, nevertheless, affirm the feasibility for the detection and identification of the RF drone database.

Based on the above works, there are three research papers that used same dataset in their implementation: in Reference [32], authors solved the detection problem using three DNNs: the first DNN was used to classify the presence of the UAV or not as a two classes problem; the second DNN was used to specify the presence of the UAV, as well as the type of the detected UAV as four classes problem; and the last DNN was used to specify the presence, type, and the mode of the detected UAV as ten classes problem. However, the main goal of this research is to show the feasibility of the dataset regardless the achieved accuracy. In the second research [26], authors tried to solve the detection problem and achieve better accuracy for the two and four classes problems compared with Reference [32] by using same structure of the three scenarios while using CNNs not DNNs. In the last research [28], authors used XGBoost algorithm for the classification problem by using only the lower band of the collected RF signals, not both bands, as in References [26,32]. However, they achieved better accuracy around 70% for the case of 10-classes compared with the other neural networks-based research studies. Table 2 summarizes some of these research studies.

## 3. Methodology

In this section, we introduce the system model that is employed to construct the RF-UAV dataset and show the feasibility of the dataset for the UAV detection and identification problem. First, we present the system model and its components. Then, we describe the detection and identification problem. Next, we describe the structure of the RF dataset and the number of segments/samples for each case.

### 3.1. System Model and UAV Detection Problem

UAVs typically include on-board transmitters to control and run the UAV using only the RF signal, which are used in the communication between the UAV and the controller for data exchange purposes. These transmitter is generally works based on the 2.4 GHz Industrial, Scientific, and Medical (ISM) band. UAVs can be observed and identified from a long distance based on both the UAV itself and the characteristic of the used receiver about the surrounding environment. In addition to the benefit of employing RF signal to detect the UAVs, the used controller to transmit the signal might also be located to allow human to locate and identify the source signal.

**Figure 1:**
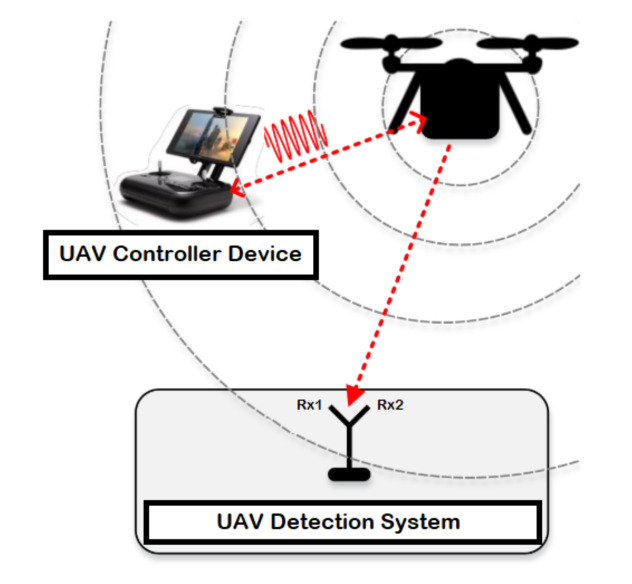
System model for UAV detection.

The detection system consists of a UAV, device to control the UAV known by controller, and two receiver units for recording the received signal strength resulted from the UAV (i.e., the first one records the lower band of the RF signals, while the second receiver captures the upper band of the RF signals) as illustrated in Figure 1. RF signals represents the communication signals between the UAV and the controller device, where the RF signals are mainly collected by employing a receiver unit (e.g., USRP-2943 40 MHz RF receiver) with an appropriate specification based on the surrounding environment as described in Table 3. At last, the collected data is fetched, stored and processed using a laptop or any other interfacing device equipped with LabVIEW software.

In this research, we will use a public dataset, where the collected data is based on multiple experiments as discussed in Reference [34]. The first component in the system model is the UAV, authors used three types of drones/UAVs: Parrot Bebop, Parrot AR Drone, and DJI Phantom 3. They are widely used in the research field, and each one has different specifications from the other, as described in Table 4. The three UAVs are mainly considered as in the small UAV category due to their weights (0.4–1.2) Kgs.

The second component in the detection system model is the controller device, which represents a controlling unit that is working based on a specific application. The main goal of this application is to send/receive the main tasks to the UAV (i.e., the mode) using the RF signals. Each of the UAVs has a different application based on the developing company in order to achieve specific tasks.

The receiver unit represents the third component in the detection system; it is used to detect the RF signals from different UAVs based on the wireless communication. The main objective of this module is to detect the transmitted information in the RF form over the wireless medium. Next, this module needs to be connected with another interfacing device for fetching, analyzing, and storing the collected RF signals directly in a predefined database.

### 3.2. Dataset

A public dataset is used to train the proposed approach for detection and idintification the UAVs, and it is generated based on multiple experiments. The experimental system as discussed in the previous subsection contains different types of UAVs, controllers, receivers, and end user. UAVs distributed to serve a specific area with random altitudes, to study the behaviour of the received signal strength for these heterogonous UAVs. ML is used for learning the detection system based on the captured RF data. For powerful classification approaches with low variance and bias values, both quantity and quality of data were considered as important factors in the training and testing scenarios. Hence, the approach should operate based on huge quantities of data to obtain sufficient information and to improve its generalization capabilities in order to get less variance values, as well as avoid the overfitting problem. On the other hand, the dataset should cover number of cases that originate from or imitate real scenarios to reduce the bias values in the learned model, as well as avoid the underfitting effect.

In UAV classification problems, reference datasets for the different modalities are still not generally known and validated. For most, if not all, researchers obtained their own data through various methods, including laboratory experiments, simulations, and outdoor measurements. In certain cases, data collected in the laboratory, for example, are combined with a certain amount of noise to simulate a real environment. This research is based on a dataset that was generated based on a real experiment [34]. It consists from set of recorded raw samples and segments for each class with respect to different number of experiments. The collected RF data at the receiver side is usually used for training and testing the model. The recorded time for the RF background activities is around 10.25 s, and the RF UAV communication for each flight mode is roughly around 5.25 s. The used dataset in validating our approach was published in 2019 [34], and the recorded segments were collected based on three different levels as illustrated below:

- **Level 1:** it represents whether a UAV is in the air or not. Hence, there are two classes to describe this level: class one: No UAV, class two: UAV.
- **Level 2:** it shows the type of the detected UAV, when there is a UAV in the air based on Level 1. We employ three classes based on how many type of UAVs in the network and we name them based on their types: class one: Parrot Bebop, class two: Parrot AR, and class three: Phantom 3.
- **Level 3:** it represents the flight mode of the UAV that was detected in Level 2. So, we have four modes (four classes) for each UAV (e.g., Parrot Bebop and Parrot AR):
  1. **Class One:** ON (mode-1).
  2. **Class Two:** Hovering (mode-2).
  3. **Class Three:** Flying (mode-3).
  4. **Class Four:** Recording video (mode-4).

**Figure 2:**
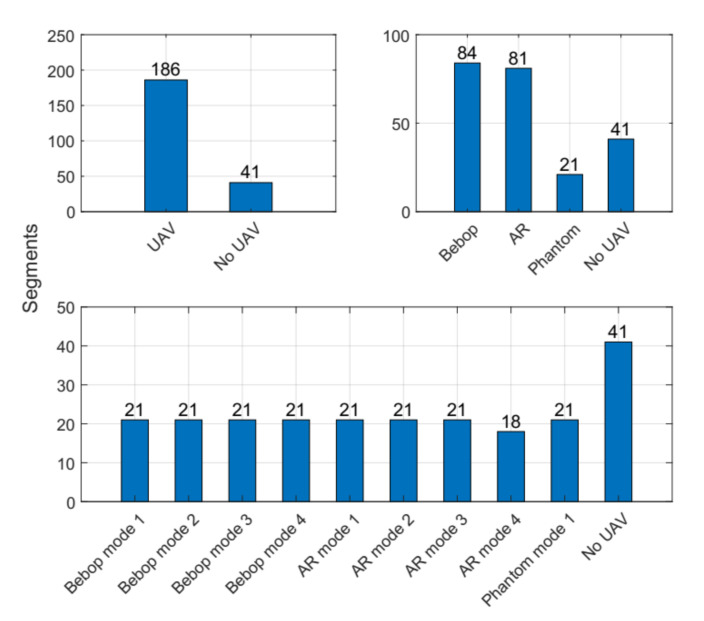
Segments for each set of classes (2, 4, and 10) at each experiment level in the public Radio Frequency (RF) dataset.

In order to prevent memory overload, the collected RF-signals were stored as segments in a CSV-format, based on the authors in Reference [34]. This makes loading and interpreting of the RF-UAV dataset using any compatible application simple and even easier than other formats. Figure 2 shows in details the recorded segments for each experimental level in the RF-UAV dataset. The dataset contains in total 227 segments, and each segment consists of 20 million samples; hence, this makes the dataset in range of a huge dataset. This dataset has been used in previous research studies [26,28,32], and the distribution of samples for all cases is as follows:

1. **Bebop UAV:** 84 segments (84 × 20 × 106 samples).
2. **AR UAV:** 81 segments (81 × 20 × 106 samples).
3. **Phantom UAV:** 21 segments (21 × 20 × 106 samples).
4. **No UAV:** 41 segments (41 × 20 × 106 samples).

Based on the above description of the dataset [34], the detection problem can be solved as a multi class problem in a hierarchical manner. From our understanding, the system will be built based on four classifiers to identify the presence of the UAV, type of the UAV, and the mode of the UAV as discussed in the next section.

## 4. Proposed Detection Approach

Based on the problem description, we propose an intelligent approach to classify each stage correctly, taking into our consideration the overall accuracy of all stages, as well as the simplicity of our approach. The first stage is the problem formulation, which mainly consists of specifying the system model and the collected data from the model in order to be investigated and analyzed in the next stages. UAV detection is a multi-class classification problem, and we consider some approaches in our design that have shown a good performance in multi-class classification scenarios. Stage two represents the pre-processing elements that are required to clean, transform, smooth, and normalize the collected data to be used in the next stages. Stage three represents the construction of our approach, where the learning approach is designed to solve this multi-class classification problem using different learning methods in a hierarchical manner. The last stage shows the evaluation process in term of different performance metrics. A lot of strategies and assumptions throughout the model construction and the data analysis stages are used to improve the performance of the detection system. This ML approach is built based on Scikit-learn libraries in Python 3 [35].

### 4.1. Data Pre-Processing Stage

**Figure 3:**
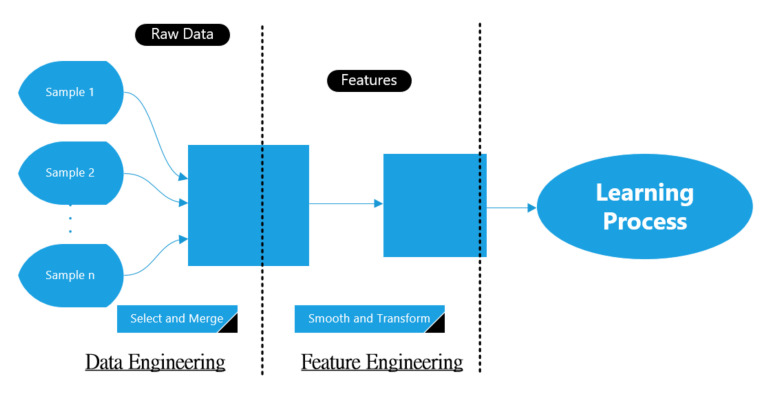
Block diagram of the data pre-processing stage.

Raw RF data in the main dataset was collected using on-board sensing units, which requires to be pre-processed again before entering the learning approach. This includes the filtering process that uses to eliminate the noises and conflict, or using various techniques to shrink the size of the data. The data pre-processing stage in ML approach consists of two units: data engineering and feature engineering. The data engineering represents how to convert the RF data from raw shape to a form to be used later on, while the feature engineering represents how to tune the resulted data from the data engineering in order to construct the features desired by the learning approach. Figure 3 shows the typical block diagram of this stage.

In reality, methods from both signal processing and ML can often be combined to increase the predictability of the model. In our scenario, a filtering stage is applied to the input samples for smoothing purposes and reducing the noise value. This filtering stage is based on averaging concept with a predefined window. It basically does the averaging by adding N-adjacent samples and then dividing the sum by N value. Next, it directly writes the values into the Nth sample position. In general, this method represents a Finite Impulse Response (FIR) filter of a specific number of taps (N) with a uniform weighting option. Finally, smoothed values enter the learning approach in order to be classified based on a given target values.

### 4.2. Binary/Multi-Class Classification Stages

Classification concept is one of the supervised ML techniques, where used samples in the training and testing processes are labeled. The number of labels for a certain dataset is the same as the number of classes. The identification and detection of UAV using ML is considered as a binary classification problem, in which there are only two labels (“UAV” and “No UAV”). The detection of UAVs from birds and UAVs from other aircrafts is also cosidered as a binary classification problem with the respective data labels. In our case, multi-class problem refers to different classes and labels as we have different types of UAVs and different fight modes.

In multi-class scenarios, each training instance classifies into one class from a set of classes. The objective is to extract a feature that correctly predicts the class to which the new point belongs in view of a new data point. There are different methods that can be used to solve the multi-class problem by deploying a set of binary classifiers. One way to do that is by constructing a classification system that can classify the raw data to ten classes (from 1 to 10) by deploying a 10 binary classifiers for each collection of data. Next, if you choose to classify a certain data point, you will obtain each classifier’s decision score for that point and select the class based on the highest classification score. This solution is known by one-versus-all strategy or one-versus-the-rest. One-versus-all strategy operates by training a binary classifier for data and then matches each classifier to each input to decide the class to which input belongs to. The steps of this strategy can be described as follows:

1. Since we have a multiclass classification problem with N classes (2, 3, and 4), the one-versus-all strategy transforms the training dataset into some N binary classification problems.
2. For each class, allocate negative dataset to the input from other classes and allocate the positive dataset for the selected class. Then, we need to train this approach using a specific classifier to fit the binary dataset.
3. After doing the training of these binary classifiers for each class, we need to specify every input that belongs to the selected class, if its hypothesis returns the highest score with respect to other classifiers.

Using the above strategy in our approach, classifying the class based on other classes, will enhance the classification accuracy for each of the following sub-classifiers in our hierarchical learning approach:

1. **Classifier 1 (Presence of the UAV):** two classes (UAV and No UAV); it is used to specify if there is a UAV or not.
2. **Classifier 2 (Type of the UAV):** three classes after detecting the presence of the UAV from the first classifier (Bebop, AR, and Phantom); it represents the presence of the UAV and the type of the detected UAV.
3. **Classifier 3 (Modes of the Bebop UAV):** four classes after detecting the type of the UAV (four flight modes for Bebop: on, hovering, flying, flying with video recording); it is used to define the presence of the UAV, the type of the detected UAV, and the flight mode of the UAV.
4. **Classifier 4 (Modes of the AR UAV):** four classes after detecting the type of the UAV (four flight modes for AR: on, hovering, flying, flying with video recording); it is used to define the presence of the UAV, the type of the detected UAV, and the flight mode of the UAV.

### 4.3. Ensemble Learning

**Figure 4:**
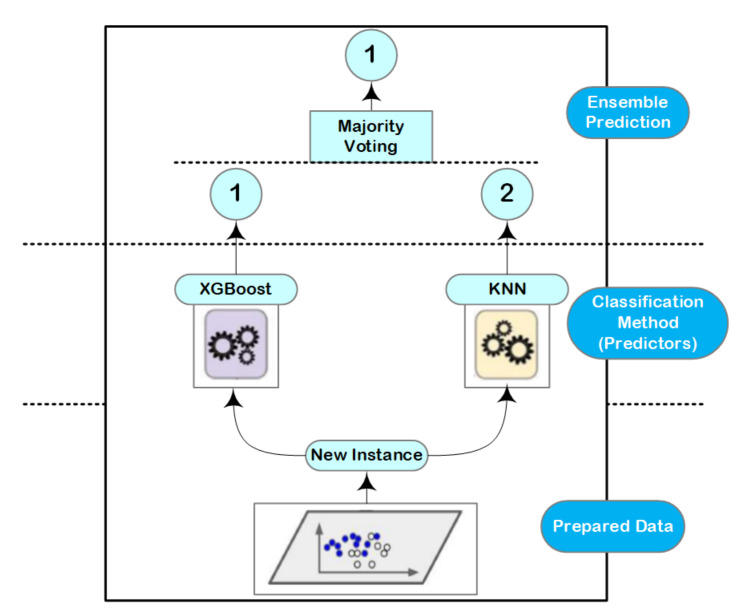
Ensemble learning approach; it consists of two learning approaches (K-Nearest Neighbor (KNN), XGBoost), along with majority voting stage to decide the output value.

Ensemble learning with decision trees concept is typically one of the highest performing approaches in case of a classification problems [36]. It basically combines different classifiers into one classifier for solving a classification problem and the final score can be selected based on the majority voting principle [37]. This voting process achieves better accuracy values compared with the best classifier in the ensemble learning as illustrated in Figure 4. Indeed, ensemble learning approach can be a strong learner even it combines multiple weak learners, given that these weak learners are sufficiently diverse.

However, the main causes of the variations between the predicted and true values are resulted from noise, bias, and variance. In our research, it can be seen that the approach is going to either overfitting or underfitting due to these factors. Using the ensemble learning helps to minimize these errors except the irreducible error (e.g., caused by noise). That is why the ensemble learning comes into the picture directly to be used in our design. The used algorithms in our approach are: XGBoost and K-Nearest Neighbor (KNN). These algorithms are suitable and can achieve better accuracy in case of multiclass scenarios. The final score of these algorithms are selected based on the majority voting of the results.

**Figure 5:**
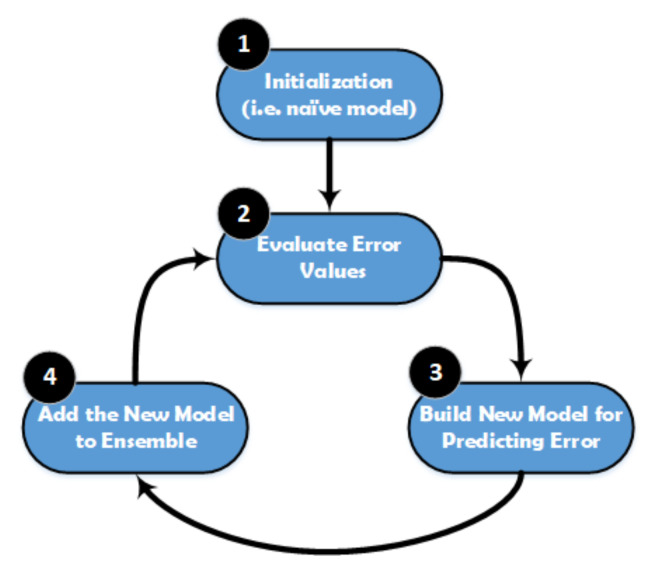
Structure of the XGBoost classifier.

XGBoost is a popular implementation of gradient boosting, and it works based on the Gradient Boosted Decision Tree model. The main steps of XGBoost method are illustrated in Figure 5. This model is initialized by using a simple base prediction, which handles the initialization of the first predictions. After that, it starts evaluating the error values for each sample in the dataset. Next, a new model is built to predict the samples based on the error values. The output predictions from the new model will be added to the ensemble of models. To predict any sample, we need to add all the predictions from the previous models and then use them to evaluate the new error value, construct a new model, and add it to the ensemble of models and so on. Some important characteristics can be achieved by using XGBoost method, especially when the dataset is large, such as regularization, cache awareness, parallel learning, and less number of splits in the trees, and the overfitting can be avoided by using a suitable stopping method for the boosting, and it has a positive impact on the out-of-core computing.

**Figure 6:**
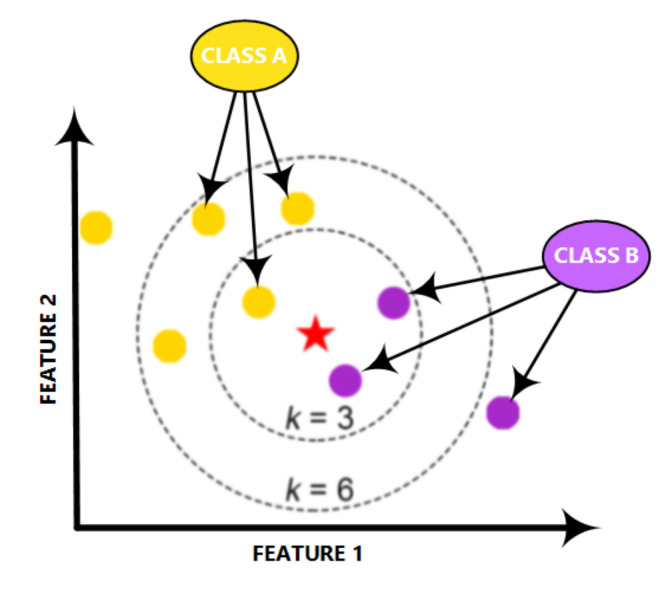
Structure of the KNN classifier.

The KNN is a powerful classifier; it is said that, for a specific value of K, the KNN algorithm finds the K nearest neighbors for a given data sample, and, next, it specifies the applicable class to this sample by choosing a class which achieves the highest number of samples out of all other classes of K neighbors, as illustrated in Figure 6.

The detection and classification problem can be solved easily in a hierarchical manner since the dataset contains RF records for a set of UAVs describing their availability, type, and mode. The hierarchical model starts by determining the availability of the UAV, and then it will specify the type of the UAV based on the detected RF signal. Once the type is defined, the mode can then be specified using the same RF signal. Therefore, it is easy to handle any sample using the hierarchical way with minimum error rate compared to a non-hierarchical approach, which warrants a need to remove the unwanted classes at each step of the classification process. The hierarchical approach minimizes the similarity of the RF signal for the detected UAVs at different classification stages. The approach consists a set of classifiers that uses the same learning process with different number of output classes (i.e., 2, 3, and 4). Each classifier works based on the ensemble learning; it is one of the best ML models that can achieve better perdition values for a set of learning techniques. Basically, the use of ensemble learning offers a systematic solution and brings a distributed model to a ML model, which helps in refining the prediction results and ensuring better accuracy. Moreover, it is an effective approach to deal with overfitting, where the model does well on the training set but fails to generalize on the test set. Since the dataset contains samples having high similarity to each other in terms of the distribution of RF signals, it is required to use a learning technique that will minimize the variance component of the prediction errors. Hence, ensemble learning is used to implement this model. It is a simple and light model, requires less processing time, and enables to find the optimal values that might reduce the variance in the RF signal with minimal processing time.

**Figure 7:**
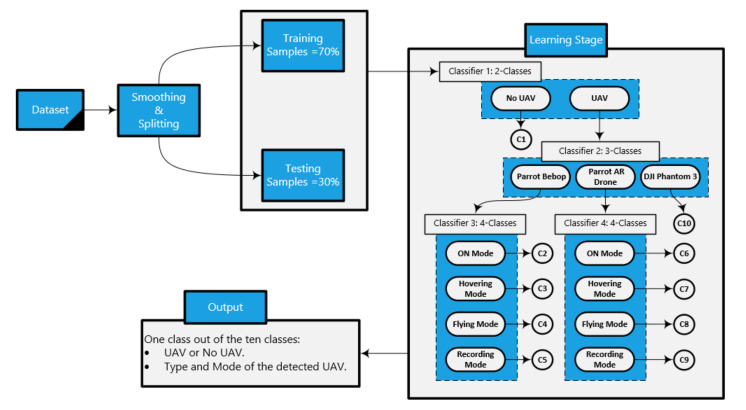
Our hierarchical learning approach consists of dividing the dataset into training and testing data stage and a learning stage with four classifiers to specify the class of any sample.

Our approach is illustrated in Figure 7, and, in Algorithm 1, the smoothed RF testing sample enters the first classifier (binary classifier) in the learning stage. The output of this classifier with respect to our RF sample informs if there is a UAV in the detected region or no UAV. If there is a UAV in the region, this sample enters the second classifier in order to define the type of the detected UAV (Bebop, AR, and Phantom 3). If the detected UAV is Phantom 3, the output of the detection approach is class number 10. Otherwise, the testing sample enters either the third classifier, and the detected UAV is *Bebop*, or the fourth classifier, when the detected UAV is *AR*. The third and fourth classifiers define the mode of the detected UAV as one of the following modes based on four classes problem: on, hovering, flying, and video recording, where the classes for the Bebop UAV are: 2 for ON mode, 3 for Hovering mode, 4 for Flying mode, and 5 for Video recording mode. On the other hand, for AR UAV, the modes are as follows: 6 for ON mode, 7 for Hovering mode, 8 for Flying mode, and 9 for Video recording mode. Each classifier works based on the ensemble concept, where the constituent model consists of XGBoost and KNN. The final output is based on the voting of the outputs from the two algorithms. These algorithms are used from the Scikit-learn libraries in Python 3, and we set the best parameters for both algorithms using the grid search model in order to achieve the best accuracy for the whole detection system.
**Algorithm 1** Hierarchical learning approach.  1:Load RF dataset  2:Define the inputs and the outputs  3:Pre-process the data  4:Encoding the outputs with respect to the inputs  5:Split the data into Sets: training (70%) and testing (30%) sets (trainSet,testSet)  6:Define the input variables and find the optimal parameters (params) using GridSearch  7:Records startTime  8:Train the system on (trainSet) by calling HierarchicalApproach procedure  9:Test the system by using (testSet) and evaluate the required metrics 10:  11:**procedure**Classifier(Sets,params) 12:  Use the optimal parameters params for KNN and XGBoost 13:  Use ensemble learning based on KNN and XGBoost 14:  Train and fit the classifier using (trainSet) 15:  Predict the test samples 16:**end procedure** 17:  18:**procedure**HierarchicalApproach(trainSet,testSet,params) 19:  Sample from Sets will pass the first Classifier to specify the availability of the UAV (2 classes: 0-No UAV, 1-UAV) 20:  **if** class==0 **then** 21:    procTime=timeNow−startTime 22:    go to end procedure and return the predicted class (predClass==C1) 23:  **else** 24:    Sample will pass the second Classifier to specify the type of the UAV (3 classes: 0-Bebop UAV, 1-AR UAV, 2-Phantom3 UAV) 25:    **if** class==2 **then** 26:      procTime=timeNow−startTime 27:      go to end procedure and return the predicted class: Phantom3 UAV (predClass==C10) 28:    **else** 29:      **if** class==0 **then** 30:       Sample will pass the third Classifier to specify the mode of the Bebop UAV (4 classes: 0-ON (C2), 1-Hovering (C3), 2-Flying (C4), 3-Recording (C5)) 31:       **if** class==0 **then** 32:         procTime=timeNow−startTime 33:         go to end procedure and return the predicted class: Bebop UAV with ON mode (predClass==C2) 34:       **else if** class==1 **then** 35:         procTime=timeNow−startTime 36:         go to end procedure and return the predicted class: Bebop UAV with Hovering mode (predClass==C3) 37:       **else if** class==2 **then** 38:         procTime=timeNow−startTime 39:         go to end procedure and return the predicted class: Bebop UAV with Flying mode (predClass==C4) 40:       **else** 41:         procTime=timeNow−startTime 42:         go to end procedure and return the predicted class: Bebop UAV with Recording mode (predClass==C5) 43:       **end if** 44:     **else** 45:       Sample will pass the fourth Classifier to specify the mode of the AR UAV (4 classes: 0-ON (C6), 1-Hovering (C7), 2-Flying (C8), 3-Recording (C9)) 46:       **if** class==0 **then** 47:         procTime=timeNow−startTime 48:         go to end procedure and return the predicted class: AR UAV with ON mode (predClass==C6) 49:       **else if** class==1 **then** 50:         procTime=timeNow−startTime 51:         go to end procedure and return the predicted class: AR UAV with Hovering mode (predClass==C7) 52:       **else if** class==2 **then** 53:         procTime=timeNow−startTime 54:         go to end procedure and return the predicted class: AR UAV with Flying mode (predClass==C8) 55:       **else** 56:         procTime=timeNow−startTime 57:         go to end procedure and return the predicted class: AR UAV with Recording mode (predClass==C9) 58:       **end if** 59:     **end if** 60:    **end if** 61:  **end if** 62:  Return predicted class (predClass) and processing time (procTime) 63:**end procedure**

## 5. Results and Discussions

This section presents the results of the developed UAV detection and identification approach. First, we present the pre-processing tasks to get a cleaned data, which is suitable to be used in the learning approach directly. Next, we show the representation of the RF data in time and frequency domain for some of the selected UAV modes. Then, we show and discuss the results of our approach for the 10-classes scenario with respect to our smoothed RF data. Finally, we compare our results with other papers that used same dataset in case of 10 classes.

### 5.1. Pre-Processing Stage

Our selected dataset [34] consists of around 227 segments of the recorded RF signals during multiple experiments. Each experiment was conducted using three type of drones/UAVs (Bebop, AR, and Phantom 3). The collected RF records contain around 10.25 s of RF background data when the UAVs are off and 5.25 s of UAVs communication RF data. Data was also recorded for different flight modes: on, hovering, only flying, and flying with video recording. The three UAVs was controlled by using a flight controllers based on their companies. However, two RF receivers (NI-USRP 2943R RF) were used for detection the RF signals that are coming from the UAVs, and both receivers are directly connected to laptop. The first one records the lower band of the RF signal, and the second one is for detecting the upper band of the RF signal. Authors in Reference [34] used the two RF receivers with a maximum instantaneous bandwidth of 40 MHz that are working simultaneously to at least record part of the spectrum as in WiFi with 80 MHz bandwidth. The exact bandwidth of 2.4 GHz-WiFi is equal to 94 MHz + 3 MHz, and the second term is used as guard bands at the start and the end. So, authors did not record the first and last 1-MHz and the last channel since they contain negligible information in addition to high noises and interferences. Therefore, the dataset has almost clean RF signals based on this strategy. Hence, we used this dataset and added the filtering stage to overcome/minimize the effect of the interferences caused by high density RF signals.

**Figure 8:**
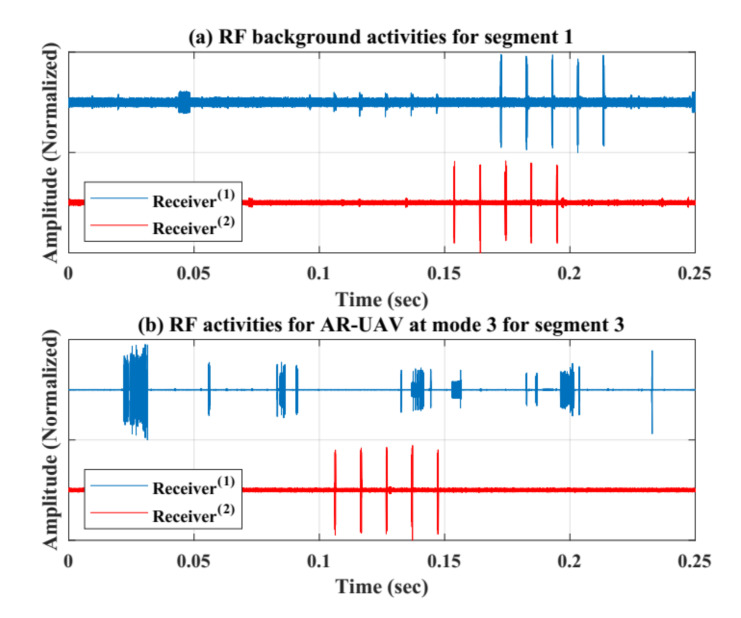
RF activities with normalized amplitudes (−1 and 1). (**a**) normal RF activities for segment 1. (**b**) RF activities for AR-UAV at mode 3 for segment 3.

The collected RF data are pre-processed by means of removing the noise for each RF signal using a smoothing filter with window equal to 15. Hence, results define that the RF signals are improved, and noises and clutters are minimized, based on reducing the variation for each data sample to be smoother than the original RF data. Furthermore, this dataset has been segmented into smaller number of segments to ensure better performance in the learning process, and these segments were represented by the Fast Fourier Transform (FFT) using MATLAB commands. Indeed, the FFT was done with zero mean signals by removing the zero frequency parts. This decreases the overall process’ computational complexity. Moreover, this stage is critical specially when the RF signals are weak in the presence of the noise and omitting the bias effect in the signals leads directly to better detection accuracy. Figure 8 shows the collected RF signals using two receivers units for two segments after doing the normalization (between 1 and −1 values). (a) presents the normal RF activities for segment 1, while (b) presents the RF activities when there is a AR UAV only flying in the region without recording videos using segment 3.

### 5.2. Analysis of RF Dataset

**Figure 9:**
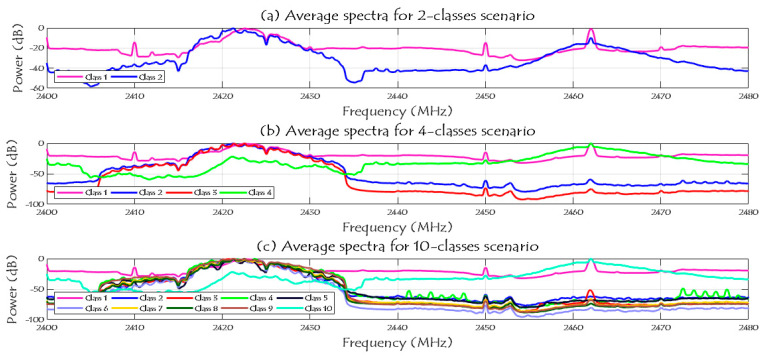
Average spectra of the RF activities for 2, 4, and 10 classes scenarios.

The main challenge here is to extract the important information from the generated data to use them as predictor in the learning process. This part identifies the main features that are considered and chosen as inputs and the explanations behind using these features in the learning approach. Figure 2 shows the specifications of the RF dataset and how many segments (=20 million samples per segment) for each class. It is shown in the same figure that each class has different number of segments/samples, and we divided our dataset into 70% for training process and 30% for testing process. The spectrum signals of the three given scenarios in the dataset (2, 4, and 10 classes) are shown in Figure 9 after reducing the noise variations and smooth the RF signals.

Note that, in case of two classes scenario, class 1 represents the RF background activities for no UAV case and class 2 represents the RF signals when there is a UAV. In case of four classes scenario, class 1 represents the RF background activities for no UAV case, and classes from 2 to 4 are for the Bebop, AR, and Phantom UAVs. In case of ten classes, class 1 represents the RF background activities for no UAV case, classes from 2 to 5 represents the flight modes of the Bebop 4, classes from 6 to 9 represents the flight modes of the AR 4, and, finally, class 10 represents the flight mode of the Phantom UAV.

**Figure 10:**
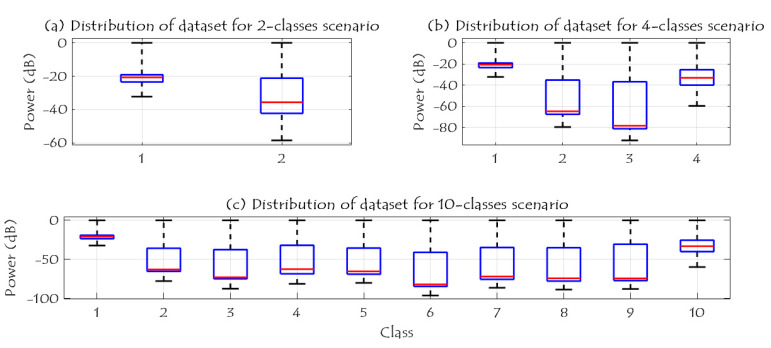
Data distribution for 2, 4, and 10 classes scenarios.

Figure 10 shows the RF data distribution of 2-classes, 4-classes, and 10-classes scenarios. Based on (a), the data distribution of the two classes (UAV and No UAV) can be easily used to identify the availability of the UAV in a specific region. Furthermore, when the number of classes has increased to four classes, the complexity of the detection problem is increased due to the same RF range for class 2 and class 3 as illustrated in (b); these two classes are the Bebop and AR UAVs that made by the same company, while class 1 and 4 can be easily identified since they are transmitted using different RF values. In case of 10 classes, class 1 and class 10 can be easily detected, while it is difficult to identify class 2 to class 5 since they have the same range of RF values for Bebop 4 flight modes, and the same thing for class 6 to class 9.

### 5.3. UAV Detection and Identification

The easiest way to test any detection system is by measuring the overall accuracy. However, in a situation where classes are not equally relevant to classify or when classes are imbalanced, accuracy can be a misleading option. Hence, the ratios of positive or negative being incorrect or correct are also more helpful in the evaluation. There are other metrics that might use to measure the performance of the UAV detection system, such as recall, error, precision, false negative rate (FNR), false discovery rate (FDR), and F1 scores. These metrics can be evaluated based on the following equations:

(1) $$\mathrm{Accuracy}=\;\frac{\mathrm{TP}+\mathrm{TN}}{\mathrm{TP}+\mathrm{TN}+\mathrm{FP}+\mathrm{FN}},$$

(2) $$\mathrm{Precision}=\;\frac{\mathrm{TP}}{\mathrm{TP}+\mathrm{FP}},$$

(3) $$\mathrm{Recall}=\;\frac{\mathrm{TP}}{\mathrm{TP}+\mathrm{FN}},$$

**Figure 11:**
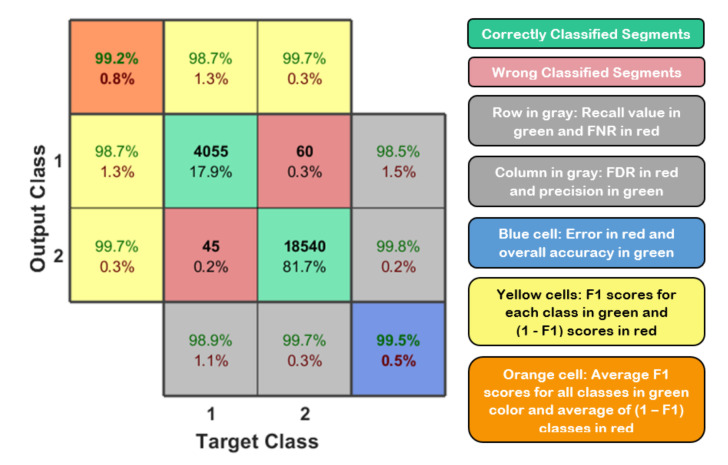
Typical confusion matrix for binary classification on the left side and the representation of these values based on their background colors on the right side [32].

(4) Error=1−Accuracy,

(5) FDR=1−Precision,

(6) FNR=1−Recall,

(7) $$F1\;\mathrm{score}=2\times \left(\frac{\mathrm{Precision}\times \mathrm{Recall}}{\mathrm{Precision}+\mathrm{Recall}}\right),$$

where TP, FN, and FP represent the number of true positives, false negatives, and false positives [38]. These metrics can be evaluated and summarized in a matrix form called confusion matrix. Figure 11 shows the typical confusion matrix for binary classification problem that can be generated and visualized using the output of a python 3 script and MATLAB code.

So, many performance metrics are needed to be considered when evaluating the proposed approach in order to correctly describe the overall performance of the detection and identification system. In this research, we mentioned the best evaluation metrics that need to be taken into account in the evaluation process for our learning approach. Performance evaluation of the learning approach is illustrated and summarized in the below confusion matrix for the 10 classes scenario. As discussed before, we solved our detection problem in Hierarchical manner using four classifiers (classifier with 2-classes, classifier with 3-classes, and two classifiers with 4-classes). Each classifier works based on ensemble concept using KNN and XGBoost classifiers. The reasons behind using these classifiers is that they are supporting regularization, flexibility, and built-in cross validation. Both classifiers have a set of hyper-parameters that need to be tuned to achieve better performance. The tuning process is done once in our model using “Grid Search with cross validation (cv = 3)”, and the output parameters of both methods are: for XGBoost (learning rate = 0.1, number of estimators = 100) and for KNN (K = 1) and we used the default values for other hyper-parameters [39]. In general, the selection of these values will affect the overall output of the ensemble learning, thereby influencing the accuracy in addition to other evaluation metrics. We used the optimal values to avoid the overfitting and underfitting problems caused by setting wrong hyper-parameters. Another performance metric can be used in evaluating the learning approach is the processing time. It represents the minimum required time for the system to correctly detect the sample. It depends on the shape and the size of the dataset, as well as the machine that will handle the detection and identification process. We can consider it as the testing time of our approach, which represented based on our simulation for all samples between 6.54 msec and 7.41 msec in all scenarios.

**Figure 12:**
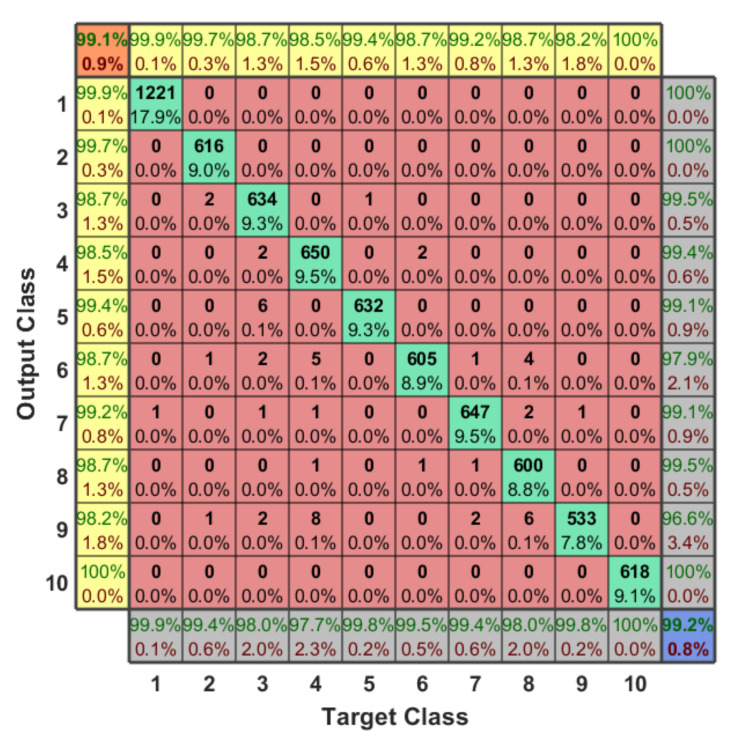
The output confusion matrix for 10-classes scenario (presence of the UAV, type of the UAV, and flight mode of the UAV).

The RF dataset has 2048 features which are fed into a hierarchical learning approach for classification. These features represent the lower and upper bands of the RF signals that collected using two receivers as explained before and in the experiment setup of Reference [34]. In our case, and based on the RF dataset, we labeled our 10 classes using hot encoding in order to solve this 10-classes problem, and each sample has a specific label with respect to our classes from 1 to 10. Therefore, the final output of any testing sample specifies the presence of the UAV, the type of the detected UAV, and the flight mode of this UAV. Figure 12 shows the confusion matrix of the 10-classes problem. The classification accuracy is 99.20%, average F1 score is 99.10%, recall is 99.11%, the average detection error is around 0.80%, and the testing time for any sample to be detected correctly is in range of 6.54 msec and 7.41 msec in all scenarios.

We can conclude from the confusion matrix of our learning approach that solving the detection problem in a hierarchical manner minimizes the error value. Since, we are dividing our problem into four small classification problems with respect to the testing sample, each classifier finds the target class of the sample and then forwards the testing sample to the next classifier, and so on, until specify the type and flight mode of the detected UAV. Based on our dataset for the first classifier, we notice that it is easy to determine the presence of the UAV since each RF data has different variations from the other one, where the error value here is almost zero (one sample is only detected wrong). Therefore, we have only two classes, either “No UAV” or “UAV”. In order to define the type of the UAV, we need to enter the second classifier. The output of this classifier is one class out of three classes (Bebop, AR, and Phantom 3). Since we have only one flight mode for the Phantom 3 based on our RF dataset, this considers directly as class 10. On the other hand, if the testing sample is not Phantom 3, it enters the next classifier (4-classes) in order to specify the flight mode based on the detected type (Bebop or AR) from previous classifier. For Bebop, we have 4 classes (from 2 to 5) and 4-classes for AR (from 6 to 9).

**Figure 13:**
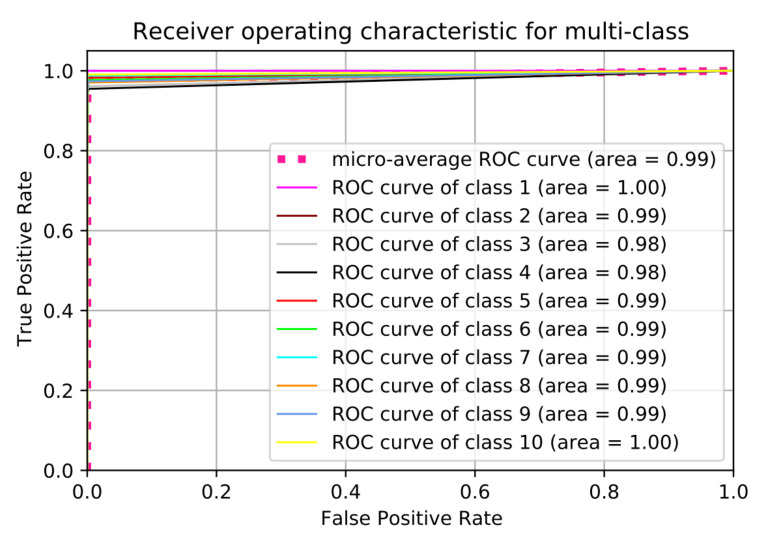
Receiver Operating Characteristic (ROC) curve for 10 classes scenario.

Somehow, we notice that the classification accuracy decreases when the number of classes increases. Class 2 to class 9 have same range of RF values (Bebop and AR) for different flight modes since they are produced by same company so that the detection accuracy is low compared with phantom UAV. On the other hand, the recall values remain high for the 10 classes with value around 99.11%. We plotted the Receiver Operating Characteristic (ROC) curve. It is a useful tool to predict the probability of a binary output. The *x*-axis of the figure represents the False Positive Rate (FPR) and the *y*-axis represents the True Positive Rate (TPR) with different probability values from 0 to 1 for the classes. Figure 13 shows the ROC, along with the Area Under the ROC curve (AUC), of our approach with 10 classes based on the optimal hyper-parameters.

## 6. Comparison with Other Approaches

We compared our approach with three papers that used same dataset in their implementation [26,28,32]. In Reference [32], authors used three layers to implement the deep learning approach and also used 10-fold cross validation concept in the training and testing stages of the learning approach, while, in Reference [26], they tried to solve the detection problem and achieve better accuracy using CNNs not DNNs. Lastly, in Reference [28], authors used XGBoost algorithm for solving the classification problem by using only the lower band of the RF signals. The performance of the four approaches are summarized in Table 5.

Our hierarchical learning approach outperforms other learning approaches in the 10 classes detection problem in terms of accuracy, F1 score, and recall. However, the findings of the developed approach also show the feasibility of using this RF dataset for identification and detection UAVs.

## 7. Conclusions and Future Work

The identification and detection problem of RF signals is studied for different type of UAVs in this paper. The hierarchical detection approach is implemented to check the availability of a UAV, specify the type of the UAV, and then determine the flight mode of the detected UAV. Further, the proposed approach is designed to work in the presence of the other noises resulting from devices that are working at same frequency band. Such noises can be minimized through the pre-processing stage by reducing the bias and variance effects from the RF signals with respect to our normal operating signal for the selected flight mode. At the time of detecting the UAV signal, RF fingerprints and hierarchical approach for classification purposes are used to detect and identify the detected signal for the targeted class. This approach shows the possibility to reach a classification accuracy around 99%. As a future work, we plan for carrying out further evaluation with more datasets, including representation of diverse types of UAVs.

## Figures and Tables

**Table 1:** Summary of the detection methods.

Method	Pros	Cons
**Radar**	Best in fog, cloud, and dust comparing to visual detection. Less noise effect comparing to acoustic detection. No need for LoS. Doppler signature option can be used for detection.	Drones has smaller cross-sections of radar that make identification more difficult. In radar system, mmWave technology has a greater path loss which limits the range of drone detection.
**Visual**	Low-cost in terms of hardware needs, e.g., cameras. Detection using screens is easier than other ways.	It is not suitable in dust, fog, cloud, and day time. High resolution cameras are needed. LoS is necessary.
**Acoustic**	No LoS. Low-cost in terms of microphone arrays.	Sensitive to ambient noise. Not suitable with wind. Needs acoustic signature dataset for training and testing.
**RF signal**	Low cost RF sensors. No LoS. Long detection range.	Not ideal for autonomous drone detection without communication channels. It needs training to learn RF signatures.

**Table 2:** Summary of the Unmanned Aerial Vehicle (UAV) detection approaches based on Machine Learning (ML).

Ref.	Type	Features	Dataset	Loss Criteria	Training Model	Measures
[15]	Binary-class classification	Angle and position	800 frames from 20 videos	Likelihood	Haar with Adaboost, Gaussian Mixture Model (GMM)	True positive and false alarm rates
[25]	Multi-class classification	Acoustic fingerprints	Public dataset, Training 70%, Validation 15%, and Testing 15%	MSE	CNN, RNN, and CRNN	CPU-Time, accuracy, recall precision, and F1-score
[33]	Multi-class classification	ADr sounds	Trained with 138 samples and tested with 34 sound samples	Error	SVM with various kernels	Accuracy
[27]	Multi-class classification	Packet size and inter-arrival time	Dataset contains 3k traffic traces, and non UAV type contains 3k traffic traces. 70% for training and 30% for testing	Mean Square Error (MSE), MLE	One-versus-all logistic regression	Computational time and accuracy
[26]	Multi-class classification	RF signatures	681 segments; each segment is $20\times 10^{6}$ samples	MSE	CNN	Accuracy recall
[28]	Multi-class classification	RF signatures	681 segments; each segment is $20\times 10^{6}$ samples	MSE	ML (XGBoost)	Accuracy
[29]	Multi-class classification	Passive RF signals, SNR	Each controller 100 RF signals, contains 5000k samples. Training (60%) + Cross-validation (20%) and 20% for Testing)	Negative Log Likelihood	Discriminant Analysis (DA), SVM, KNN, and NN	Accuracy, false alarm rate
[30]	Binary-class classification	RF signals	Windows with dimension of 200×3276×2	MAE	CNN	MAE
[31]	Multi-class classification	RF fingerprints	100 RF signals for each of 14 UAV controllers, 80% of data for training and 20% for testing	Entropy	KNN, DA, SVM, and NN	Accuracy
[32]	Multi-class classification	RF signatures	681 segments; each segment is $20\times 10^{6}$ samples	MSE	Three DNN	Accuracy recall

**Table 3:** Features of USRP-2943 RF receiver [34].

Specification	Value
Number of channels	2
Frequency range	[1.2–6] GHz
Frequency step	<1 KHz
Gain range	[0–37.5] dB
Maximum instantaneous bandwidth	40 MHz
Maximum I/Q sample rate	200 MS/s
ADC resolution	14 bits

**Table 4:** Features of the selected UAVs/drones.

UAV/Drone	Parrot Bebop	Parrot AR	DJI Phantom 3
Dimensions (cm)	38×33×3.6	61×61×12.7	52×49×29
Weight (Kgs)	0.400	0.420	1.216
Battery capacity (mAh)	1200	1000	4480
Maximum range (m)	250	50	1000
Connectivity	WiFi (2.4 & 5 GHz)	WiFi (2.4 GHz)	WiFi ([2.4–2.483] GHz) RF ([5.725–5.825] GHz)

**Table 5:** Comparison between our approach and other related approaches using same dataset.

Reference	ML Approach	Accuracy (%) for 10-Classes
[26]	CNN	59.2
[28]	XGBoost	70.1
[32]	DNN	46.8
ours	Hierarchical	99.2

## Data Availability

The data used in this study are available online under this website https://data.mendeley.com/datasets/f4c2b4n755/1 accessed on 1 November 2020.

## Abbreviations

The following abbreviations are used in this manuscript:

UAVs	Unmanned Aerial Vehicles
RF	Radio Frequency
LiDaR	Light Detection and Ranging
RADAR	RAdio Detection And Ranging
LoS	Line-of-Sight
CNN	Convolutional Neural Network
ANN	Artificial Neural Network
NN	Neural Network
XGBoost	eXtreme Gradient Boosting
RNN	Recurrent Neural Network
CRNN	Convolutional Recurrent Neural Network
TCT	Target Candidates Track
ADr	Amateur Drone
MFCC	Mel Frequency Cepstral Coefficients
SVMs	Support Vector Machines
MLE	Maximum Likelihood Estimation
NLoS	Non-Line of Sight
NCA	Neighborhood Component Analysis
DNNs	Deep Neural Networks
DFT	Discrete Fourier transform
D-STFT	Discrete-Short Time Fourier Transform
GMM	Gaussian Mixture Model
KNN	K Nearest Neighbour
ISM	Industrial, Scientific, and Medical
FIR	Finite Impulse Response
FNR	False Negative Rate
FDR	False Discovery Rate
FFT	Fast Fourier Transform
MSE	Mean Square Error
MAE	Maximum Absolute Error
DL	Deep Learning
DA	Discriminant Analysis
FPR	False Positive Rate
TPR	True Positive Rate
ROC	Receiver Operating Characteristic
AUC	Area Under ROC Curve
